# Supplementary material for: The Role of Hidden Conformers in Determination of Conformational Preferences of Mefenamic Acid by NOESY Spectroscopy
Source: Pharmaceutics. 2022 Oct 24;14(11):2276. doi: 10.3390/pharmaceutics14112276 (PMC9696638; doi:10.3390/pharmaceutics14112276)
Supplement: Supplementary file 1 [file pharmaceutics-14-02276-s001.zip › pharmaceutics-1980007-supplementary.pdf]

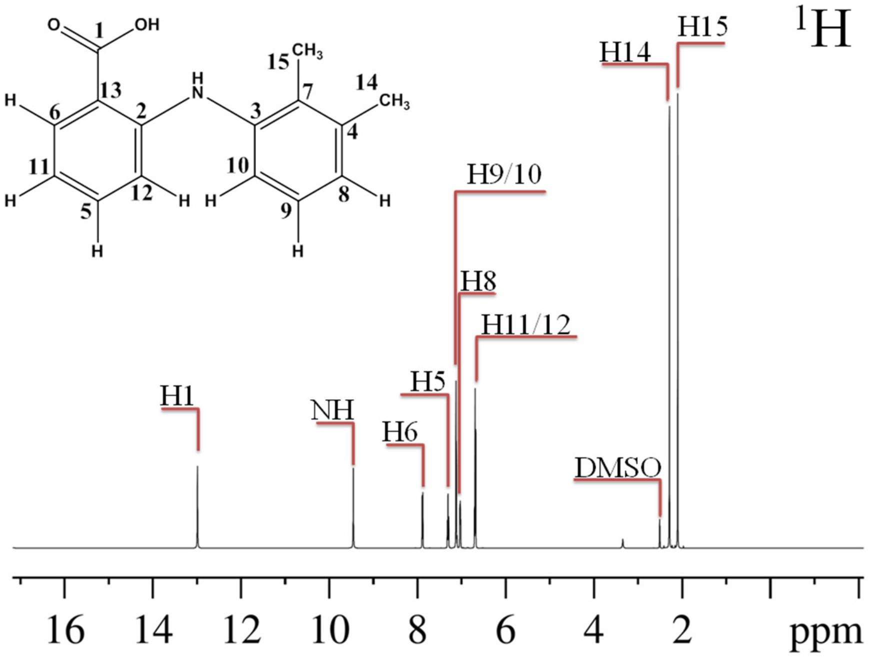

Figure S1. The ^{1}H NMR spectrum of MFA in DMSO-d6.

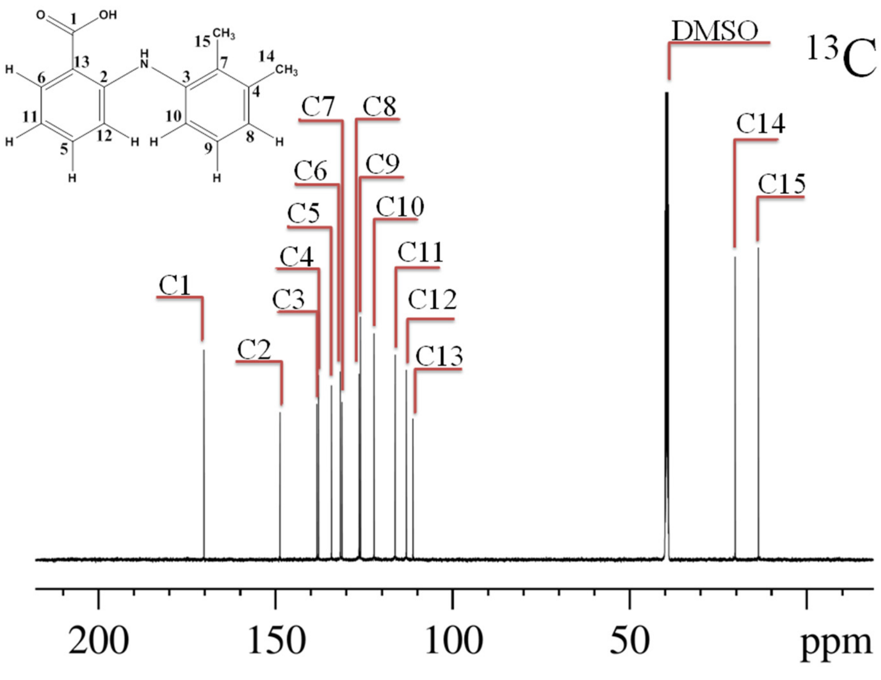

Figure S2. The ^{13}C NMR spectrum of MFA in DMSO-d6.

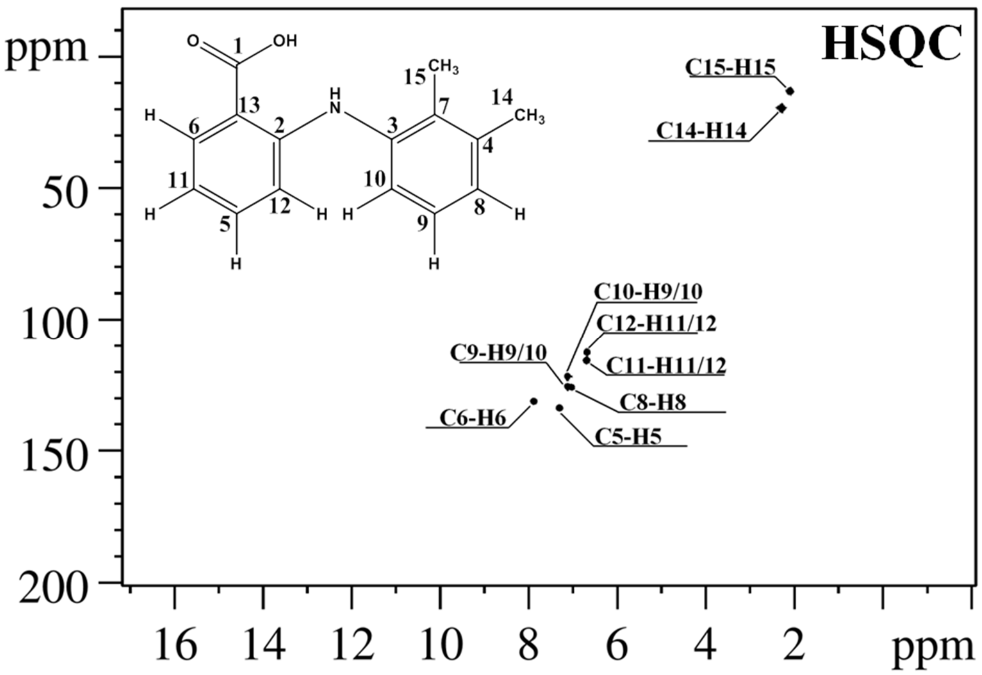

Figure S3. The ^{1}H-^{13}C HSQC spectrum of MFA in DMSO-d_6.

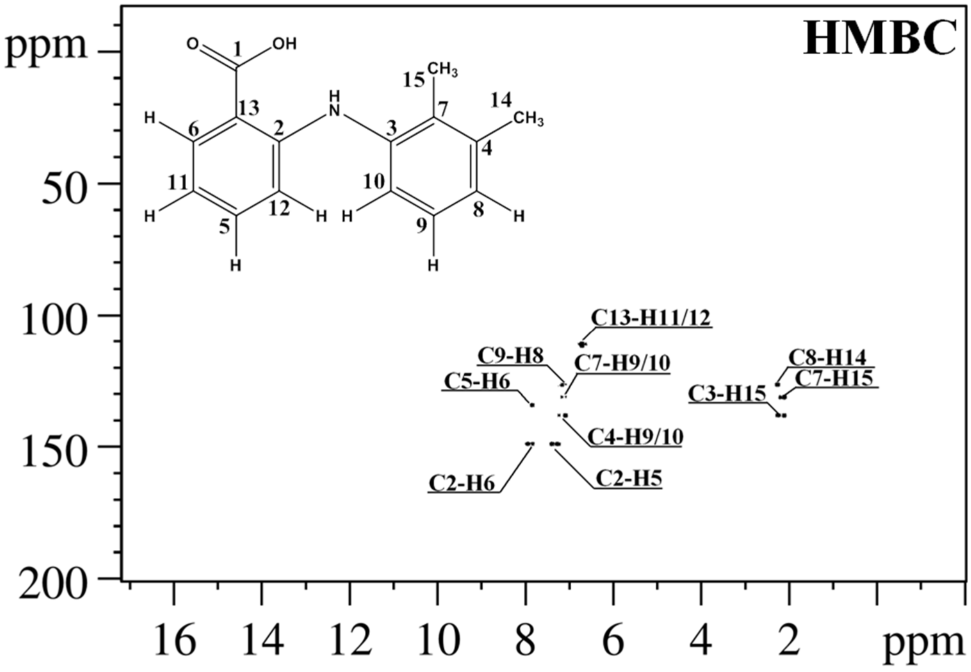

Figure S4. The ^{1}H-^{13}C HMBC spectrum of MFA in DMSO-d_6.

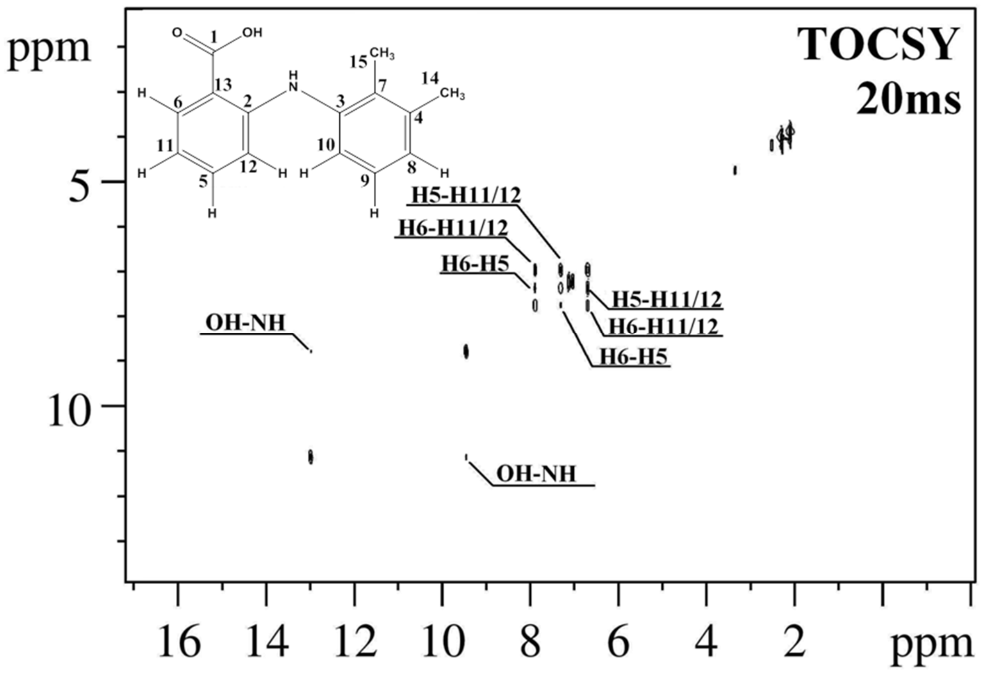

Figure S5. The ^{1}H-^{1}H TOCSY spectrum (spin-lock 20 ms) of MFA in DMSO-d$_{6}$.

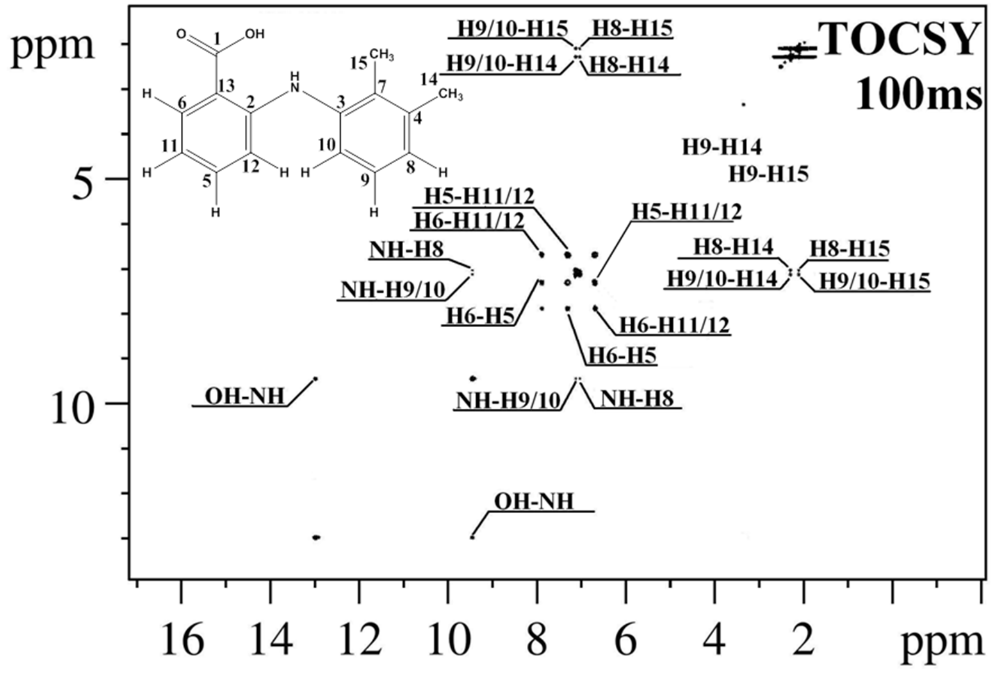

Figure S6. The ^{1}H-^{1}H TOCSY spectrum (spin-lock 100 ms) of MFA in DMSO-d$_{6}$.

<table>
<tr><td rowspan="2" colspan="2"></td><td colspan="3">**Fragment**</td></tr>
<tr><td>**-COOH (OH-H6)**</td><td>**Rings (H9/10-H11/12)**</td><td>**-OH (OH-H6)**</td></tr>
<tr><td rowspan="4">**Solvent**</td><td>**DMSO-d$_6$ (25 °C, 0.1 MPa)**</td><td>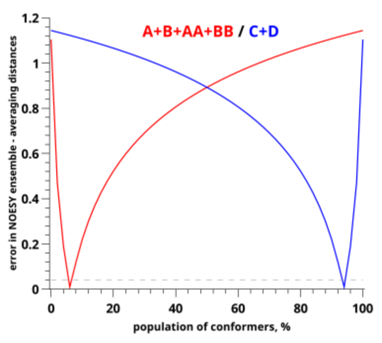
</td><td>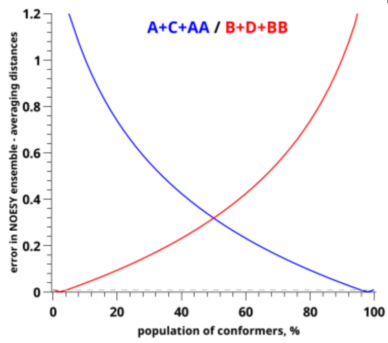
</td><td>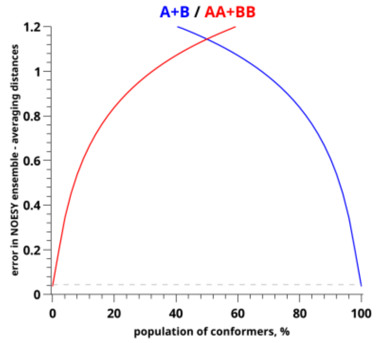
</td></tr>
<tr><td></td><td>(a)</td><td>(b)</td><td>(c)</td></tr>
<tr><td>**scCO$_2$+DMSO-d$_6$ (45 °C, 9 MPa)**</td><td>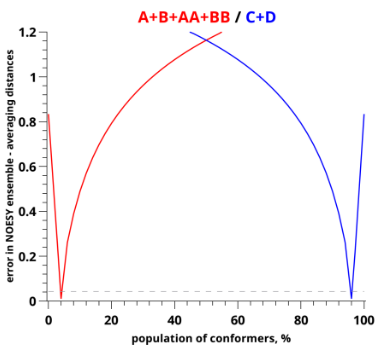
</td><td>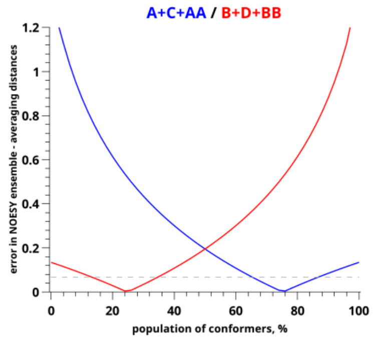
</td><td>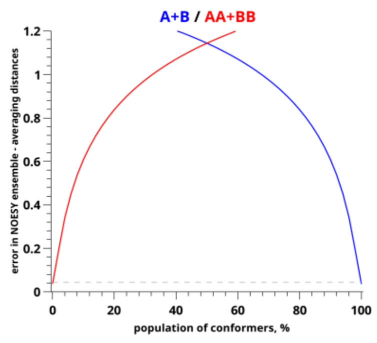
</td></tr>
<tr><td></td><td>(d)</td><td>(e)</td><td>(f)</td></tr>
</table>

Figure S7. The plots of populations of the conformer groups (blue and red curves) of MFA in (**a**–**c**) DMSO-d$_6$ and (**d**–**f**) $scCO_2$+DMSO; dashed gray line shows the error level of determining the distances.

Table S1. The chemical shift of the ^{1}H and ^{13}C nuclei is determined by 2D spectra of the MFA molecule in DMSO-d_6.

δ 13C	1H	δ 1H	HMBC	HSQC	NOESY	TOCSY		^{13}C
						20мс	100мс	
170.18	OH	12.99	-	-	OH-H15<br>OH-H9/10<br>OH-H6<br>OH-NH	-	-	C1
148.73	NH	9.45	C2-H5	-	NH-H15<br>NH-H11/12<br>NH-H9/10<br>NH-H6	OH-HN	OH-HN	C2
138.32	-	-	C3-H10	-		-	HN-H9/10	C3
137.86	-	-	C3-H15	-		-	HN-H8	C4
134.17	H5	7.31	-	C5-H5	H5-H11/12	H5-H6	H5-H6	C5
131.68	H6	7.88	-	C6-H6	H6-H11/12	H5-H11/12	H5-H11/12	C6
131.22	-	-	C7-H15	-		H6-H11/12	H6-H11/12	C7
126.39	H8	7.03	C8-H14	C8-H8	H8-H14	-	H8-H14	C8
126.00	H9/10	7.12	-	C9-H9	H9/10-H11/12	-	H8-H15	C9
122.17			-	C10-H10		-	H9/10-H14	C10
116.22	H11/12	6.69	-	C11-H11	H11/12-H15	-	H9/10-H15	C11
113.05			-	C12-H12		-	-	C12
111.19	-	-	C13-H12	-		-	-	C13
20.19	H14	2.29	C14-H15	C14-H14		-	-	C14
13.64	H15	2.10	-	C15-H15		-	-	C15

Table S2. Normalized integral intensities of the cross-peaks, cross-relaxation rates, and proton–proton distances in the MFA molecule used to calculate the conformer's populations in DMSO-d_6.

Groups	r_{calc}, Å	Dist.	Mixing Time	Relative Integral Intensity	Cross-Relaxation Rate, s^{-1}	r_{exp}, Å
A-BB	2.76	H6-H11/12 (Ref)	0.15	0.0047	$3.34 \pm 0.02 \times 10^{-2}$	-
			0.2	0.0065		
			0.25	0.0085		
			0.3	0.0103		
			0.35	0.0117		
			0.4	0.0134		
			0.45	0.015		
			0.5	0.0167		
			0.55	0.0183		
			0.6	0.0197		
			0.65	0.0216		
			0.7	0.0231		
			0.75	0.0255		
			0.8	0.0264		
			0.85	0.0283		
			0.9	0.0302		
A + C + AA	3.12	H9/10-H11/12	0.15	0.0022	$1.57 \pm 0.02 \times 10^{-2}$	3.13 ± 0.009
			0.2	0.0028		
			0.25	0.0044		
			0.3	0.0046		
			0.35	0.0055		
			0.4	0.0064		
			0.45	0.0071		

			0.5	0.0079		
B + D + BB	4.62		0.55	0.0086		
			0.6	0.0096		
			0.65	0.0101		
			0.7	0.0108		
			0.75	0.0123		
			0.8	0.0124		
			0.85	0.0136		
			0.9	0.0137		
A + B + AA + BB	2.09	OH-H6	0.15	0.0028	1.28 ± 0.09 × 10^{-2}	3.24 ± 0.04
			0.2	0.0034		
			0.25	0.0044		
			0.3	0.0052		
			0.35	0.0057		
			0.4	0.0065		
			0.45	0.0072		
			0.5	0.0075		
C + D	4.34		0.55	0.0086		
			0.6	0.0093		
			0.65	0.0098		
			0.7	0.0101		
			0.75	0.0107		
			0.8	0.0098		
			0.85	0.0117		
			0.9	0.0122		
A + B	3.27	OH-H6	0.15	0.0028		
			0.2	0.0034		
			0.25	0.0044		
			0.3	0.0052		
			0.35	0.0057		
			0.4	0.0065		
			0.45	0.0072		
			0.5	0.0075		
AA + BB	1.88		0.55	0.0086		
			0.6	0.0093		
			0.65	0.0098		
			0.7	0.0101		
			0.75	0.0107		
			0.8	0.0098		
			0.85	0.0117		
			0.9	0.0122		

Table S3. Normalized integral intensities of the cross-peaks, cross-relaxation rates, and proton–proton distances in the MFA molecule used to calculate the conformer's populations in $scCO_2$ + DMSO-d_6 (45 °C; 9 MPa).

Groups	r_{calc}, Å	Dist.	Mixing Time	Relative Integral Intensity	Cross-Relaxation rate, s^{-1}	r_{exp}, Å
A-BB	2.76	H6-H11/12 (Ref)	0.5	0.0175	$4.18 \pm 0.3 \times 10^{-2}$	-
			0.6	0.0222		
			0.7	0.0263		
			0.8	0.0336		
			0.9	0.0379		
A + C + AA	3.12	H9/10-H11/12	0.5	0.0073	$1.56 \pm 0.09 \times 10^{-2}$	3.25 ± 0.06
			0.6	0.0082		
B + D + BB	4.62		0.7	0.0101		
			0.8	0.0125		
			0.9	0.0143		
A + B + AA + BB	2.09	OH-H6	0.5	0.0041	$0.99 \pm 0.08 \times 10^{-2}$	3.51 ± 0.09
			0.6	0.0048		
C + D	4.34		0.7	0.0064		
			0.8	0.0075		
			0.9	0.0092		
A + B	3.27	OH-H6	0.5	0.0041		
			0.6	0.0048		
AA + BB	1.88		0.7	0.0064		
			0.8	0.0075		
			0.9	0.0092		

Table S4. The energy value, structure, coordinates of atoms, and the values of their magnetic shielding obtained from the data of quantum chemical calculations for conformer A MFA.

Conformer A Energy [B3LYP/6-311 + G(2d,p)]: −786.103161016 Hartrees

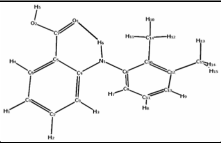

NMR Atom Assignment	Number of Atom	Type of Atom	Cartesian Coordinates X	Y	Z	GIAO Magnetic Shielding Value Isotropic	Anisotropy
C11	1	C	3.5077230	1.9514650	0.6797400	64.2483	161.7342
C5	2	C	2.2283320	2.4990980	0.8189910	43.4392	188.7689
C12	3	C	1.0987960	1.7585120	0.5402800	67.0328	158.4338
C2	4	C	1.1920860	0.4197070	0.1028500	26.6988	170.5302
C13	5	C	2.4972220	−0.1472500	−0.0191290	70.8070	134.6337
C6	6	C	3.6249480	0.6401880	0.2703430	44.4374	186.7397
H11	7	H	4.3885410	2.5407160	0.8986340	25.1479	3.8846
H5	8	H	2.1135490	3.5216180	1.1607460	24.4827	5.7578
H12	9	H	0.1214140	2.1994580	0.6743540	24.6500	11.4082
H6	10	H	4.6021200	0.1895660	0.1679030	23.6297	7.8532
C1	11	C	2.6739520	−1.5418890	−0.4280800	7.1983	97.6875
=O	12	O	1.7797190	−2.3520730	−0.6286820	−51.7522	453.8324
-O	13	O	3.9690560	−1.9184970	−0.5799740	124.2022	180.4740
OH	14	H	3.9442030	−2.8522640	−0.8382270	26.4385	9.8351
N	15	N	0.0766940	−0.3285300	−0.1619900	136.0226	78.3520
NH	16	H	0.2697490	−1.3064430	−0.3546160	22.1525	18.0949
C3	17	C	−1.2579580	0.1270820	−0.2988950	38.2300	141.2724
C10	18	C	−1.5480210	1.2238830	−1.1146230	56.4677	168.4255
C7	19	C	−2.2917900	−0.5818670	0.3388780	44.9491	172.3756

C9	20	C	−2.8594060	1.6312150	−1.2897250	53.0539	176.2622
H10	21	H	−0.7416110	1.7359760	−1.6236060	24.3796	9.5310
C4	22	C	−3.6233960	−0.1758310	0.1316760	37.8531	189.0063
C8	23	C	−3.8891260	0.9312900	−0.6721350	52.7418	149.6283
H9	24	H	−3.0819720	2.4803810	−1.9249270	24.5962	4.9966
H8	25	H	−4.9166330	1.2428820	−0.8221040	24.7011	6.4024
C15	26	C	−1.9729140	−1.7659280	1.2172390	168.4083	16.8768
H15	27	H	−2.0045160	−2.7065820	0.6556600	29.9402	9.1541
	28	H	−0.9752070	−1.6805820	1.6470460	29.3678	6.8475
	29	H	−2.6858300	−1.8602410	2.0364660	29.7736	7.5039
C14	30	C	−4.7703300	−0.9190810	0.7724340	159.8300	30.8109
H14	31	H	−4.7671910	−1.9789830	0.5038860	29.5277	7.5918
	32	H	−4.7286640	−0.8670210	1.8645230	29.3440	7.3841
	33	H	−5.7257240	−0.4977680	0.4583720	29.7723	7.7549

Table S5. The energy value, structure, coordinates of atoms, and the values of their magnetic shielding obtained from the data of quantum chemical calculations for conformer B MFA.

Conformer B Energy [B3LYP/6-311 + G(2d,p)]: −786.102589748 Hartrees

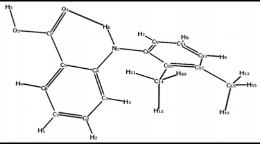

NMR Atom Assignment	Number of Atom	Type of Atom	Cartesian Coordinates			GIAO Magnetic Shielding Value	
			X	Y	Z	Isotropic	Anisotropy
C11	1	C	2.9830990	2.2977990	−0.6241520	64.7947	160.8037
C5	2	C	1.6226860	2.5248980	−0.8551150	42.5810	190.0385
C12	3	C	0.6882480	1.5290980	−0.6592950	67.1245	155.3384
C2	4	C	1.0718340	0.2451870	−0.2166360	24.4906	175.4423
C13	5	C	2.4624130	0.0057390	0.0014120	71.7664	133.1066
C6	6	C	3.3838970	1.0462540	−0.2052390	44.4893	186.761
H11	7	H	3.7091890	3.0851330	−0.7786310	25.1714	3.9868
H5	8	H	1.2892510	3.4974290	−1.1995960	24.5051	6.4230
H12	9	H	−0.3570330	1.7220600	−0.8579700	25.6542	11.7156
H6	10	H	4.4312470	0.8425950	−0.0316090	23.6107	7.7653
C1	11	C	2.9364920	−1.3150270	0.4174650	6.9231	97.1448
=O	12	O	2.2440810	−2.3101550	0.5772720	−53.6010	458.9161
-O	13	O	4.2764900	−1.3861930	0.6258700	124.9274	182.2667
OH	14	H	4.4550590	−2.3031700	0.8835540	26.4667	9.8436
N	15	N	0.1431820	−0.7344940	−0.0015530	140.3582	49.0369
NH	16	H	0.5304810	−1.6544220	0.1827190	22.1043	18.3819
C3	17	C	−1.2558870	−0.6239300	−0.2544230	39.0878	137.9923
C10	18	C	−1.7754240	−1.2893280	−1.3659050	52.1812	156.4795
C7	19	C	−2.0995130	0.0882890	0.6138410	39.4743	180.6434
C9	20	C	−3.1342140	−1.2458060	−1.6332110	51.7108	178.2366
H10	21	H	−1.0986210	−1.8308210	−2.0157310	24.6499	8.1194
C4	22	C	−3.4762570	0.1472080	0.3209420	37.8056	190.5075
C8	23	C	−3.9736900	−0.5211590	−0.7961880	50.2867	154.0110
H9	24	H	−3.5365940	−1.7639750	−2.4953910	24.5255	4.8012
H8	25	H	−5.0358080	−0.4753200	−1.0097960	24.5137	6.5231
C15	26	C	−1.5600630	0.7649190	1.8473320	167.7335	19.3317
H15	27	H	−2.1749970	0.5335610	2.7199340	30.0479	8.6267
	28	H	−0.5404310	0.4505870	2.0581760	29.3921	7.7883
	29	H	−1.5575510	1.8545410	1.7407300	30.0503	7.1951
C14	30	C	−4.4197070	0.9136270	1.2144550	160.3062	30.9767
H14	31	H	−4.4507770	0.4934570	2.2243060	29.4676	7.6731

	32	H	−4.1193980	1.9601520	1.3176150	29.5706	7.2196
	33	H	−5.4343470	0.8938120	0.8157310	29.7215	7.9856

Table S6. The energy value, structure, coordinates of atoms, and the values of their magnetic shielding obtained from the data of quantum chemical calculations for conformer C MFA.

Conformer C Energy [B3LYP/6-311 + G(2d,p)]: −786.097060464 Hartrees

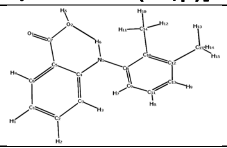

NMR Atom Assignment	Number of Atom	Type of Atom	Cartesian Coordinates			GIAO Magnetic Shielding Value	
			X	Y	Z	Isotropic	Anisotropy
C11	1	C	3.4899430	1.9817550	0.6754480	62.6384	163.9127
C5	2	C	2.2002480	2.4968710	0.8271970	43.6798	187.4218
C12	3	C	1.0903340	1.7227510	0.5597810	66.0500	158.763
C2	4	C	1.2084380	0.3863280	0.1218180	29.5040	167.5947
C13	5	C	2.5241950	−0.1466710	−0.0140000	67.7752	128.559
C6	6	C	3.6308060	0.6739110	0.2646590	41.3379	192.9403
H11	7	H	4.3596560	2.5908200	0.8843580	25.0371	3.9408
H5	8	H	2.0593330	3.5157980	1.1697570	24.4933	5.8561
H12	9	H	0.1031310	2.1381900	0.7035200	24.6797	11.6312
H6	10	H	4.6117310	0.2340570	0.1435480	23.3638	7.4962
C1	11	C	2.8697580	−1.5189730	−0.4337430	11.3293	76.3155
=O	12	O	3.9895100	−1.9260560	−0.6417170	−64.9048	471.7770
-O	13	O	1.8038360	−2.3727000	−0.5757000	120.9487	187.6655
OH	14	H	2.1821620	−3.2206650	−0.8556410	25.9262	9.9952
N	15	N	0.0797920	−0.3613310	−0.1258710	137.6461	61.5659
NH	16	H	0.2314170	−1.3457460	−0.2788710	24.2498	16.4077
C3	17	C	−1.2467650	0.1099580	−0.2923660	37.6662	141.6637
C10	18	C	−1.5114810	1.1859290	−1.1437120	56.4680	169.0132
C7	19	C	−2.2981420	−0.5644230	0.3540770	45.6508	170.9799
C9	20	C	−2.8149060	1.6049370	−1.3469250	52.5997	177.3379
H10	21	H	−0.6915720	1.6740420	−1.6546390	24.3453	9.6474
C4	22	C	−3.6218710	−0.1486210	0.1171860	37.8969	188.4835
C8	23	C	−3.8621360	0.9369750	−0.7229340	52.6558	149.8958
H9	24	H	−3.0179480	2.4383800	−2.0088690	24.6053	5.0612
H8	25	H	−4.8831890	1.2577670	−0.8954970	24.6936	6.3911
C15	26	C	−2.0037810	−1.7222360	1.2755130	168.5504	16.8615
	27	H	−2.0168810	−2.6801510	0.7421850	29.9907	9.0947
H15	28	H	−1.0201230	−1.6194410	1.7339480	29.3787	6.5142
	29	H	−2.7406360	−1.7960740	2.0750500	29.6967	7.5380
C14	30	C	−4.7871270	−0.8557320	0.7659920	159.8281	30.8623
	31	H	−4.7961470	−1.9235640	0.5310520	29.5489	7.6227
H14	32	H	−4.7590220	−0.7691730	1.8563330	29.3351	7.4141
	33	H	−5.7323020	−0.4314390	0.4262440	29.7635	7.7547

Table S7. The energy value, structure, coordinates of atoms, and the values of their magnetic shielding obtained from the data of quantum chemical calculations for conformer D MFA.

Conformer D Energy [B3LYP/6-311 + G(2d,p)]: −786.096817760 Hartrees

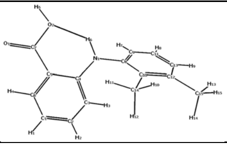

NMR Atom Assignment	Number of Atom	Type of Atom	Cartesian Coordinates			GIAO Magnetic Shielding Value	
			X	Y	Z	Isotropic	Anisotropy
C11	1	C	2.9522520	2.3276680	−0.6307440	63.118	163.3419
C5	2	C	1.5883820	2.5243020	−0.8570660	42.9201	188.6323
C12	3	C	0.6806020	1.5044660	−0.6551540	66.7143	156.1176
C2	4	C	1.0889150	0.2286000	−0.2129330	27.1560	172.3265
C13	5	C	2.4838390	0.0202850	−0.0014100	68.5298	128.0695
C6	6	C	3.3765740	1.0843770	−0.2119910	41.2493	193.257
H11	7	H	3.6627500	3.1285860	−0.7879540	25.0853	4.0394
H5	8	H	1.2287010	3.4870600	−1.2024420	24.5136	6.5212
H12	9	H	−0.3689300	1.6748580	−0.8510020	25.6954	11.9633
H6	10	H	4.4257070	0.8880940	−0.0351050	23.3329	7.4129
C1	11	C	3.1193690	−1.2412080	0.4256860	10.9575	76.9835
=O	12	O	4.2940280	−1.3833260	0.6773910	−63.9567	471.8753
-O	13	O	2.2730800	−2.3177890	0.5188710	120.3058	186.2024
OH	14	H	2.8262010	−3.0624710	0.8016550	25.8898	10.1477
N	15	N	0.1490530	−0.7504740	0.0121000	142.4738	40.4811
NH	16	H	0.5052230	−1.6866100	0.1265360	24.0171	16.7474
C3	17	C	−1.2488020	−0.6287940	−0.2489190	38.9057	138.3220
C10	18	C	−1.7699990	−1.2899840	−1.3622460	52.8779	154.8584
C7	19	C	−2.0902560	0.0878650	0.6178320	39.2970	180.8962
C9	20	C	−3.1283260	−1.2404410	−1.6314700	51.5564	178.3103
H10	21	H	−1.0954420	−1.8314210	−2.0148270	24.6558	8.1312
C4	22	C	−3.4659480	0.1546430	0.3219340	37.4616	190.9199
C8	23	C	−3.9653150	−0.5116740	−0.7957480	50.2450	154.1284
H9	24	H	−3.5316810	−1.7555410	−2.4950190	24.5086	4.8304
H8	25	H	−5.0267320	−0.4600220	−1.0112710	24.5068	6.5161
C15	26	C	−1.5488470	0.7623250	1.8514310	167.7302	19.4532
H15	27	H	−2.1682020	0.5382060	2.7227720	30.0428	8.6586
	28	H	−0.5321250	0.4404870	2.0649450	29.3635	7.6642
	29	H	−1.5368270	1.8515650	1.7422520	30.0571	7.1654
C14	30	C	−4.4067230	0.9270430	1.2130210	160.2984	30.9814
H14	31	H	−4.4410010	0.5088860	2.2235700	29.4683	7.6774
	32	H	−4.1012310	1.9721760	1.3146180	29.5641	7.2237
	33	H	−5.4209430	0.9115460	0.8131420	29.7160	7.9965

Table S8. The energy value, structure, coordinates of atoms, and the values of their magnetic shielding obtained from the data of quantum chemical calculations for conformer AA MFA.

Conformer AA Energy [B3LYP/6-311 + G(2d,p)]: −786.093686558 Hartrees

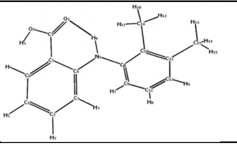

NMR Atom Assignment	Number of Atom	Type of Atom	Cartesian Coordinates			GIAO Magnetic Shielding Value	
			X	Y	Z	Isotropic	Anisotropy
C11	1	C	−3.5448970	1.8974770	−0.7085350	64.0498	161.8697
C5	2	C	−2.2787520	2.4778360	−0.8192790	44.4476	188.0788
C12	3	C	−1.1377190	1.7541480	−0.5436480	64.6512	163.2319

C2	4	C	−1.2038540	0.4038050	−0.1377670	27.9030	168.4245
C13	5	C	−2.4968050	−0.1886540	−0.0036850	66.7740	133.8085
C6	6	C	−3.6322440	0.5792860	−0.3088210	51.1475	169.562
H11	7	H	−4.4372180	2.4603250	−0.9483190	25.1424	3.9705
H5	8	H	−2.1833390	3.5069620	−1.1458980	24.4860	5.6624
H12	9	H	−0.1682660	2.2150670	−0.6679480	24.4655	11.4482
H6	10	H	−4.6151560	0.1212580	−0.2742520	24.4045	7.2132
C1	11	C	−2.6313230	−1.5983090	0.4244890	9.5176	91.7982
=O	12	O	−1.7353760	−2.4171880	0.3875240	−75.6431	548.7127
-O	13	O	−3.8388060	−2.0041010	0.8944910	134.9635	135.2242
OH	14	H	−4.4267070	−1.2485740	1.0163550	25.0559	8.3083
N	15	N	−0.0730560	−0.3332900	0.0908100	136.3198	98.0519
NH	16	H	−0.2444570	−1.3298240	0.1826490	22.0646	17.3735
C3	17	C	1.2489830	0.1363260	0.2705320	38.0528	143.2825
C10	18	C	1.5074490	1.2313440	1.0989630	58.8236	167.1311
C7	19	C	2.3058160	−0.5621390	−0.3421810	45.7568	171.2882
C9	20	C	2.8106090	1.6491410	1.3088680	53.0922	176.3116
H10	21	H	0.6855560	1.7332870	1.5929810	24.4374	9.5518
C4	22	C	3.6273510	−0.1455550	−0.0991610	37.9473	188.3545
C8	23	C	3.8620010	0.9618240	0.7150360	52.9773	148.9065
H9	24	H	3.0090290	2.4971640	1.9535250	24.6135	5.0493
H8	25	H	4.8822510	1.2824220	0.8923600	24.7313	6.4112
C15	26	C	2.0103450	−1.7463220	−1.2289020	168.4157	17.0826
H15	27	H	1.9132450	−2.6718990	−0.6499010	29.8258	9.2355
	28	H	1.0733990	−1.6091170	−1.7700060	29.4718	6.2248
	29	H	2.8001390	−1.9088240	−1.9608760	29.6575	7.4925
C14	30	C	4.8002430	−0.8747250	−0.7094200	29.3254	7.3742
H14	31	H	4.8066790	−1.9332160	−0.4359180	29.7905	7.6929
	32	H	4.7854540	−0.8266920	−1.8022280	64.0498	161.8697
	33	H	5.7410150	−0.4378930	−0.3732540	44.4476	188.0788

Table S9. The energy value, structure, coordinates of atoms, and the values of their magnetic shielding obtained from the data of quantum chemical calculations for conformer BB MFA.

Conformer BB Energy [B3LYP/6-311 + G(2d,p)]: −786.092908265 Hartrees

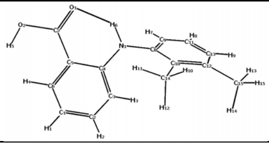

NMR Atom Assignment	Number of Atom	Type of Atom	Cartesian Coordinates			GIAO Magnetic Shielding Value	
			X	Y	Z	Isotropic	Anisotropy
C11	1	C	2.9939610	2.2668470	−0.6785870	64.8592	160.7723
C5	2	C	1.6315270	2.5131840	−0.8637190	43.4400	189.3400
C12	3	C	0.6951270	1.5230580	−0.6509550	65.2059	158.7814
C2	4	C	1.0757660	0.2289080	−0.2359870	24.9579	174.3607
C13	5	C	2.4658040	−0.0238350	−0.0237900	68.6757	132.8166
C6	6	C	3.3858510	1.0085280	−0.2668550	51.0374	169.6375
H11	7	H	3.7291500	3.0375400	−0.8682770	25.1933	4.0708
H5	8	H	1.2997460	3.4907080	−1.1944840	24.5002	6.3684
H12	9	H	−0.3525530	1.7255040	−0.8259240	25.5611	11.8032
H6	10	H	4.4495270	0.8156740	−0.1734540	24.4408	7.1753
C1	11	C	2.9200180	−1.3622000	0.4135280	9.6745	91.1977
=O	12	O	2.2409880	−2.3676500	0.3679900	−73.3708	554.4830
-O	13	O	4.1822490	−1.4763030	0.9021850	137.4400	135.8186
OH	14	H	4.5816550	−0.6058760	1.0194460	24.9594	8.2499
N	15	N	0.1432190	−0.7502150	−0.0341270	140.2205	68.506

NH	16	H	0.5314900	−1.6843260	0.0547260	21.8408	18.2506
C3	17	C	−1.2574050	−0.6215590	−0.2654000	39.179	138.6282
C10	18	C	−1.7991700	−1.2709650	−1.3760100	52.7470	156.2713
C7	19	C	−2.0814770	0.0895280	0.6223290	40.4494	179.3266
C9	20	C	−3.1607320	−1.2098680	−1.6240640	51.4369	178.6023
H10	21	H	−1.1379510	−1.8141110	−2.0404340	24.6274	8.0643
C4	22	C	−3.4609910	0.1677440	0.3476390	37.7005	190.5081
C8	23	C	−3.9807320	−0.4833500	−0.7695470	50.3796	153.8457
H9	24	H	−3.5802890	−1.7156470	−2.4853730	24.5029	4.8277
H8	25	H	−5.0449010	−0.4232810	−0.9686810	24.5178	6.5177
C15	26	C	−1.5184910	0.7419290	1.8584540	167.8345	19.7490
H15	27	H	−2.1356950	0.5206670	2.7318010	30.0303	8.6078
	28	H	−0.5074120	0.3966230	2.0627930	29.4386	7.6764
	29	H	−1.4854480	1.8321430	1.7613700	30.0654	7.1221
C14	30	C	−4.3844360	0.9348230	1.2613150	160.2410	31.0133
H14	31	H	−4.4112980	0.5010450	2.2655070	29.4505	7.6888
	32	H	−4.0689590	1.9757090	1.3754830	29.5756	7.2098
	33	H	−5.4032180	0.9341130	0.8728850	29.7187	7.9782

Table S10. Structure, coordinates of atoms, and the values of their magnetic shielding obtained from the data of quantum chemical calculations for TMS.

TMS

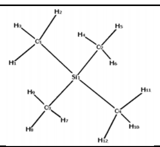

Number of Atom	Type of Atom	Cartesian Coordinates			GIAO Magnetic Shielding Value	
		X	Y	Z	Isotropic	Anisotropy
1	Si	0.0001	−0.0001	0.0001	385.7967	0.0000
2	C	−0.9231	−0.1714	−1.6233	192.5440	5.3805
3	C	0.8883	1.6510	0.0427	192.5440	5.3805
4	C	1.2519	−1.3874	0.1568	192.5440	5.3805
5	C	−1.2172	−0.0922	1.4236	192.5440	5.3805
6	H	−1.4495	−1.1294	−1.6692	32.0644	9.8877
7	H	−1.6598	0.6296	−1.7351	32.0644	9.8877
8	H	−0.2308	−0.1203	−2.4688	32.0644	9.8877
9	H	1.6044	1.7260	−0.7809	32.0644	9.8877
10	H	0.1752	2.4758	−0.0476	32.0644	9.8877
11	H	1.4342	1.7720	0.9831	32.0644	9.8877
12	H	1.8026	−1.3064	1.0986	32.0644	9.8877
13	H	0.7543	−2.3614	0.1340	32.0644	9.8877
14	H	1.9729	−1.3521	−0.6653	32.0644	9.8877
15	H	−0.6990	0.0058	2.3822	32.0644	9.8877
16	H	−1.9578	0.7099	1.3513	32.0644	9.8877
17	H	−1.7473	−1.0491	1.4175	32.0644	9.8877
